# Expression of the immune regulation antigen CD70 in osteosarcoma

**DOI:** 10.1186/s12935-015-0181-5

**Published:** 2015-03-18

**Authors:** Jens HW Pahl, Susy J Santos, Marieke L Kuijjer, Gerharda H Boerman, Laurens GL Sand, Karoly Szuhai, Annemarie Cleton-Jansen, R Maarten Egeler, Judith VMG Boveé, Marco W Schilham, Arjan C Lankester

**Affiliations:** Department of Pediatrics, Leiden University Medical Center, 2333ZA Leiden, The Netherlands; Department of Pathology, Leiden University Medical Center, 2333ZA Leiden, The Netherlands; Department of Molecular Cell Biology, Leiden University Medical Center, 2333ZA Leiden, The Netherlands; Innate Immunity Group, German Cancer Research Center, 69120 Heidelberg, Germany; Division of Hematology/Oncology, Hospital for Sick Children/University of Toronto, M5G1X8 Toronto, Canada

**Keywords:** CD70, Osteosarcoma, Immunotherapy, CD27

## Abstract

Osteosarcoma is the most frequent bone cancer in children and young adults. The outcome of patients with advanced disease is dismal. Exploitation of tumor-immune cell interactions may provide novel therapeutic approaches. CD70-CD27 interactions are important for the regulation of adaptive immunity. CD70 expression has been reported in some solid cancers and implicated in tumor escape from immunosurveillance. In this study, expression of CD70 and CD27 was analyzed in osteosarcoma cell lines and tumor specimens.

CD70 protein was expressed on most osteosarcoma cell lines (5/7) and patient-derived primary osteosarcoma cultures (4/6) as measured by flow cytometry. In contrast, CD70 was detected on few Ewing sarcoma cell lines (5/15) and was virtually absent from neuroblastoma (1/7) and rhabdomyosarcoma cell lines (0/5). CD70^+^ primary cultures were derived from CD70^+^ osteosarcoma lesions. CD70 expression in osteosarcoma cryosections was heterogeneous, restricted to tumor cells and not attributed to infiltrating CD3^+^ T cells as assessed by immunohistochemistry/immunofluorescence. CD70 was detected in primary (1/5) but also recurrent (2/4) and metastatic (1/3) tumors. CD27, the receptor for CD70, was neither detected on tumor cells nor on T cells in CD70^+^ or CD70^−^ tumors, suggesting that CD70 on tumor cells is not involved in CD27-dependent tumor-immune cell interactions in osteosarcoma. CD70 gene expression in diagnostic biopsies of osteosarcoma patients did not correlate with the occurrence of metastasis and survival (n = 70).

Our data illustrate that CD70 is expressed in a subset of osteosarcoma patients. In patients with CD70^+^ tumors, CD70 may represent a novel candidate for antibody-based targeted immunotherapy.

## Introduction

Osteosarcoma is the most frequent bone cancer in children and adolescents. Despite intensive chemotherapy, patients with recurrent, metastatic and chemotherapy-refractory osteosarcoma have a poor prognosis [1]. Osteosarcoma is frequently infiltrated by immune cells such as macrophages and T cells [2,3]. Insight in tumor-immune cell interactions may be instrumental to develop novel treatment approaches like targeted immunotherapy. Aside from its role in adaptive immunity, CD70 is expressed on certain solid tumors and reported to be involved in tumor cell escape from immunosurveillance [4].

CD70 is the natural ligand for the tumor necrosis factor (TNF) superfamily member CD27 and has originally been described as a co-stimulatory molecule for B cell and T cell activation [4,5]. Expression of CD70 in non-malignant tissue is primarily confined to cells of the hematopoietic system, yet mostly transient and tightly regulated [4-7]. CD70 is absent from human and murine naïve T cells, immature B cells and Natural Killer (NK) cells, but induced by T cell and B cell receptor triggering, B cell transformation or NK cell activation by interleukin-15 (IL-15) [4,8]. While absent from immature dendritic cells and neutrophils [9], CD70 expression can be induced on myeloid and plasmacytoid dendritic cells by e.g. toll-like receptor ligands in combination with CD40 ligation [10,11]. Interaction of CD70 with its receptor CD27 has originally been demonstrated to enhance the expansion, interferon-γ (IFN-γ) and IL-2 production and alloreactive cytotoxicity of T cells [12,13]. In addition, CD70 promotes survival of T cells responding to low-affinity or low-dose antigens [14,15].

Accordingly, CD27 is expressed on naïve and central memory CD4 and CD8 T cells as well as on thymocytes [4,13]. In addition, CD27 is expressed on mature B cells and CD70-CD27 signaling results in B cell expansion, differentiation into plasma cells and IgG synthesis [5,16]. In innate immunity, CD70-CD27 interactions induce proliferation and IFN-γ production (but not direct cytotoxicity) of NK cells *in vitro* and have been shown to mediate NK cell-dependent tumor rejection in mice [17,18]. CD27 is expressed on nearly all murine NK cells [17]. On human NK cells CD27 is acquired during maturation in lymphoid organs but down-regulated in terminal maturation stages; in peripheral blood CD27^+^ NK cells are functionally closely related to CD56^bright^ NK cells, whereas CD27^−^ NK cells correspond to CD56^dim^ NK cells [8].

In addition to its function and transient expression limited to innate and adaptive immune cells, abundant CD70 expression has been documented in B cell malignancies and renal cell carcinoma [6,19]. Hence, CD70-CD27 interactions may possess additional functions in cancer cells such as triggering tumor progression or escape from immunosurveillance [20,21]. In addition, CD70 on cancer cells is an attractive candidate for targeted immunotherapy due to its restricted expression on non-malignant cells.

In this study, we sought to determine the expression of CD70 and CD27 in osteosarcoma as well as other (pediatric) solid cancers, and the correlation with clinical outcome.

## Methods

### Patient samples

Tumor samples derived from biopsies (obtained at the time of diagnosis, pre-chemotherapy) and resections of primary, local recurrent and metastatic tumors (all post-chemotherapy) from ten high-grade osteosarcoma patients were freshly frozen in 2-methylbutane at the Department of Pathology, Leiden University Medical Center.

From five of these patients, six primary osteosarcoma cell cultures (cell passages ranging from 5 to 20) were generated from the tumor material as previously described [22]. An overview of tumor samples and primary cultures as well as clinicopathological details of osteosarcoma patients is summarized in Table 1. Tumor specimens were obtained and analyzed according to the ethical guidelines of the national organization of scientific societies (FEDERA, http://www.federa.org/gedragscodes-codes-conduct-en). CD70 gene expression was analyzed from a genome-wide gene profiling data base consisting of diagnostic biopsies of 83 high-grade osteosarcoma patients as previously published [2] (accessible online at http://hgserver1.amc.nl/cgi-bin/r2/main.cgi).

**Table 1:** **CD70 expression and clinicopathological details of patient material**

**Patient code**	**Primary tumor biopsy**			**Primary tumor resection**			**Local relapse resection**			**Pulmonary metastasis resection**			**Patient clinicopathological data of primary tumor**			
	**Code**	**Tissue CD70**	**Cell line CD70**	**Code**	**Tissue CD70**	**Cell line CD70**	**Code**	**Tissue CD70**	**Cell line CD70**	**Code**	**Tissue CD70**	**Cell line CD70**	**Sex**	**Age**	**Primary tumor site**	**Primary histological subtype**
88	L1372	+	n/a										M	16	femur	telangiectatic
363							L2792	-	-				M	17	femur	osteoblastic
369							L2531	+	+				F	31	humerus	fibroblastic
398	L2635	n/a	+										F	14	femur	conventional
404				L2826	+	+	L3312	+	+				M	46	humerus	conventional
407				L2857	n/a	-							M	9	tibia	chondroblastic
1				L47	-	n/a				L1021	-	n/a	M	25	humerus	n/a
6				L458	-	n/a	L1072	-	n/a				F	45	femur	periosteal/juxtacortical
29				L1045	-	n/a				L1020	-	n/a	M	23	femur	telangiectatic
47				L1046	-	n/a				L437	+	n/a	M	28	tibia	n/a

n/a, not available; +, positive for CD70 expression; −, negative for CD70 expression.

### Cell lines

Established osteosarcoma cell lines HOS, HOS-143b, OHS, OSA (SJSA-1), SAOS-2, U2-OS, ZK-58; and Ewing sarcoma cell lines A673, CADO-ES, ET10, EW3, IOR/BER, RD-ES, SK-ES-1, SK-N-MC, STA-ET1, STA-ET2.1, TC71, VH64 and WE68 were obtained from the EuroBoNeT cell line repository [23]. The Ewing sarcoma cell line IOR/BER was kindly provided by K. Scotlandi (Rizzoli Orthopaedic Institute, Bologna, Italy), L1062 [22] and TC32 was obtained from the American Type Culture Collection (TC32; Manassas, VA, USA). TC71 cells were cultured in IMDM medium (Invitrogen, Carlsbad, CA, USA). supplemented with 10% heat-inactivated fetal calf serum (FSC), 100 U/ml penicillin and 100 ug/ml streptomycin (P&S) (all Invitrogen). All other osteosarcoma and Ewing sarcoma cell lines as well as the EBV-transformed B-LCL cell line 107 (established in our laboratory) were cultured in RPMI-1640 medium (Invitrogen) supplemented with FCS and P&S. Ewing sarcoma cell lines were grown in 0.1% gelatin coated tissue culture flasks. The neuroblastoma cell lines SJNB8, SKNFI, SKNBE, IMR32 (obtained from ATCC) were cultured in DMEM Glutamax I medium (high glucose) supplemented with FCS, P&S and 1% MEM-non-essential amino acids (Invitrogen); UKF NBL1 and UKF NBL4 (kindly provided by U. Koehl, Medical University of Hannover, Germany) in IMDM medium with FCS and P&S; and CHP126 (obtained from ATCC) in RPMI medium with FCS and P&S. The rhabdomyosarcoma cell lines RD (obtained from ATCC) and A204, TE671, RH30 and RH41 (obtained from DSMZ, Braunschweig, Germany) were cultured in DMEM medium with the above indicated supplements). All cell lines were negative for mycoplasma infection.

### CD70 protein expression by flow cytometry

The following mouse anti-human monoclonal antibodies and mouse isotype control antibodies were used: CD70^PE^ (Ki-24) (BD Biosciences, Franklin Lakes, NJ, USA) (this antibody produced comparable results to 2F2); CD70 2F2 (0.2 μg/ml; kindly provided by R.A.W. van Lier) and IgG1 (0.2 μg/ml; R&D Systems, Minneapolis, MN, USA) followed by the goat-anti mouse Ig^APC^ secondary antibody (BD Biosciences). FACS measurements were performed with the FACSCalibur (BD Biosciences) and analyzed with the “BD Cell Quest ProTM” software (version 5.2.1). On CD70^+^ high-passage established tumor cell lines and low-passage primary tumor cultures, CD70 was homogenously expressed on the entire cell population, enabling quantification of the fluorescence intensity by the fold change of the geometric mean fluorescence intensity (geoMFI) as indicated in the FACS histogram plots. The geoMFI fold change was calculated by dividing the geoMFI of specific CD70 antibody staining by the geoMFI of the isotype control staining.

### CD70 gene expression

CD70 gene expression was analyzed from genome-wide gene profiling data of osteosarcoma cell lines and patient’s tumor specimens as previously published [2].

### Immunohistochemistry (IHC) and Immunofluorescence (IF)

Sections of 4 μm of representative tumor cryosections of resection specimens (Table 1) and of B-cell lymphoma control tissue were fixed in acetone at −20°C for 10 min (for IHC: supplemented with 0.3% hydrogen peroxide (Sigma-Aldrich, St. Louis, MO, USA) to inactivate endogeneous peroxidase), followed by incubation in 10% normal goat serum (Dako, Glostrup, Denmark) in PBS buffer to block non-specific antibody binding.

Immunohistochemical expression of CD70 was assessed using the mouse monoclonal anti-CD70 2 F2 (IgG1, 0.16 μg/ml) antibody followed by a polyclonal goat anti-mouse/rabbit/rat IgG HRP-linker antibody conjugate (Brightvision, DPVO-110HRP; Immunologic, Duiven, the Netherlands) and DAB + Substrate Chromogen System (Dako) detection. All sections were examined with an Olympus BX41 microscope and Cell^B acquisition software (Olympus, Tokyo, Japan).

Immunofluorescent double-staining for CD3 and CD70 or CD3 and CD27 was performed with rabbit polyclonal anti-human CD3 (2.4 μg/ml; Dako), CD70 2 F2 and mouse monoclonal anti-human CD27 137B4 (IgG1, 1:200; Novocastra, Leica Microsystems, Wetzlar, Germany) followed by goat anti-rabbit Alexa 488 or goat anti-mouse IgG1 Alexa 546 (1:300; Invitrogen, Carlsbad, CA, USA). All sections were examined with a Leica DM5000 fluorescence microscope and LAS-AF acquisition program (Leica, Solms, Germany).

### Statistical analysis

Statistical analyses were performed with Graphpad Prism version 5.04 (La Jolla, CA, USA). A P-value of <0.05 was considered statistically significant.

## Results

### Osteosarcoma cell lines exhibit highest expression of CD70 among pediatric solid cancer cell lines

**Figure 1:**
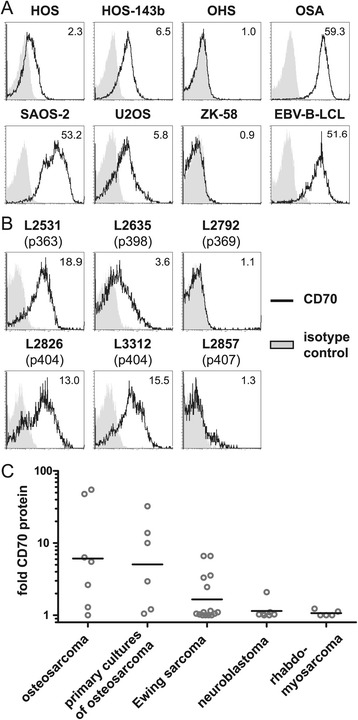
**CD70 protein is abundantly expressed on osteosarcoma cells.** CD70 protein expression on the surface of **(A)** established osteosarcoma cell lines and EBV-transformed B cell lymphoma (EBV-B-LCL) cells and **(B)** patient-derived primary osteosarcoma cultures (Table 1) was analyzed by flow cytometry. Fold change of geoMFI of specific antibody stainings (bold solid line) compared to geoMFI of isotype control (light shade) is indicated in the representative FACS histogram plots. **(C)** CD70 protein expression (fold change geoMFI, calculated as geoMFI of specific antibody staining divided by geoMFI of the isotype control staining) on the surface of established osteosarcoma cell lines and patient-derived primary osteosarcoma cultures (geometric mean of three experiments), and of established Ewing sarcoma (geometric mean of two experiments), neuroblastoma (one experiment) and rhabdomyosarcoma (one experiment) cell lines. In addition to the indicated osteosarcoma cell lines, CD70 expression was detected on the Ewing sarcoma cell lines CADO-ES, ET10, STA-ET2.1, VH64, WE68 and the neuroblastoma line CHP126.

CD70 membrane protein expression was investigated on established osteosarcoma, Ewing sarcoma, neuroblastoma and rhabdomyosarcoma cell lines by flow cytometry. Expression of CD70 was detected on five out of seven established osteosarcoma cell lines, on HOS, HOS-143b, OSA, SAOS-2 and U2OS cells but not OHS or ZK-58 cells (Figure 1, panel A). The cell line OSA exhibited the highest expression of CD70 amongst all cell lines tested which was of similar intensity as detected on EBV-transformed B cells (EBV-B-LCL). In addition, four out of six primary osteosarcoma cultures derived from five osteosarcoma patients (Table 1) were positive for CD70 expression (Figure 1 panel B).

Noteworthy, of one patient (patient (p) 404) with cultures from consecutive tumors, the culture derived from the local recurrent tumor exhibited equally high CD70 expression as the culture derived from the primary tumor of this patient. The level of CD70 expression on the primary cultures was similar to the CD70 levels on established osteosarcoma cell lines (Figure 1, panel C). In contrast to CD70, its receptor CD27 was not detected on any of the osteosarcoma cell lines (data not shown). Moreover, CD70 was detected on few established Ewing sarcoma cell lines (5/15) at lower intensities than on osteosarcoma cell lines, while CD70 was hardly detected on neuroblastoma (1/7) and rhabdomyosarcoma (0/5) cell lines (Figure 1, panel C).

### CD70^+^ primary osteosarcoma cultures are generated from parental tumors containing CD70^+^ cells

**Figure 2:**
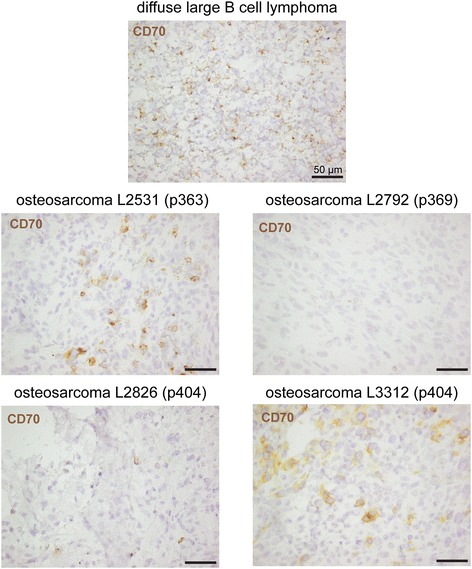
**Heterogeneous CD70 protein expression in osteosarcoma lesions.** Immunohistochemical CD70 protein staining in one specimen of diffuse large B cell lymphoma and four osteosarcoma specimens of three osteosarcoma patients (L2531 of p363; L2792 of p369; L2826 and L3312 of p404) (Table 1) Brown-colored CD70 staining patterns and counterstaining with Mayer’s hematoxylin are displayed at a 40-fold magnification.

Since in particular most osteosarcoma cell lines and patient-derived primary osteosarcoma cultures exhibited high CD70 protein expression, it was examined whether CD70 was also expressed in (corresponding) osteosarcoma tumors. Detection of CD70 by immunohistochemistry in large tissue microarrays is hampered by the lack of commercially available anti-CD70 antibodies suitable for paraffin-embedded tissue [24]. Instead, CD70 detection in individual frozen specimens is feasible as tested and described for large diffuse B cell lymphoma tissue (Figure 2) [19]. Therefore, we analyzed and compared CD70 expression in available frozen specimens of our collection of parental tumor specimens (n = 4) and corresponding primary cultures (Table 1). In addition, we compared CD70 expression between recurrent/metastatic lesions and primary tumors of the same patients (n = 5) (Table 1).

Overall, CD70 expression tended to be more frequently detected in recurrent (2/4) and metastatic (1/3) tumors than in primary osteosarcoma tumors (1/5) (Table 1). CD70 was heterogeneously expressed in the tumors in a membranous pattern (Figure 2). Of the tumor-primary culture combinations, parental tissue of L2531 (p363) exhibited CD70-expressing cells in focal regions. The high CD70 intensity of these cells was consistent with the strong CD70 expression of the corresponding primary culture (Figure 1, panel B). Of L2792 (p369), CD70 was neither detected in the parental tissue nor on the primary culture. Notably, of the one patient (p404) with tissue and primary cultures from consecutive tumors, a majority of cells of the local recurrent tumor (L3312) showed strong CD70 expression, whereas only a few cells were weakly positive in the primary tumor (L2826). Of this patient, the primary cultures of both tumors were strongly positive for CD70. In the residual four patients with combinations of consecutive tumor specimens, one metastatic tumor (L437, p47) contained CD70-expressing cells, while there were no CD70-expressing cells detectable in the primary tumor (L1046) of the same patient (Table 1).

Altogether, these results indicate that CD70 protein can be expressed in primary, recurrent and metastatic osteosarcoma lesions. Both high-passage cell lines and low-passage primary tumor cultures showed homogenous but different CD70 expression levels (or were negative) which did not seem to change after further culturing (data not shown). CD70 expression was only detected on primary cultures if these were derived from a tumor specimen containing CD70^+^ cells, indicating that CD70 expression was not caused by *in vitro* cell culture. Thus because cell lines were homogenously positive for CD70 even if they grew from tumors in which not all cells expressed CD70, these results suggest that CD70^+^ cells in the tumor preferentially grow out to CD70^+^ primary patient-derived cultures.

### CD70 expression in osteosarcoma lesions is confined to tumor cells and does not influence patient survival

To determine whether CD70 expression on tumor cells would be associated with clinical outcome of patients with osteosarcoma, we needed to investigate CD70 expression levels in a large cohort of patients with data on follow-up. For this purpose, we wanted to use a public dataset on gene (mRNA) expression of a large collection of osteosarcoma biopsies.

**Figure 3:**
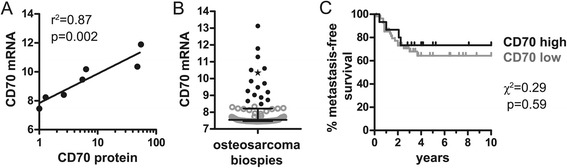
**CD70 gene expression in osteosarcoma lesions does not influence survival. (A)** CD70 protein expression (fold change geoMFI) on the surface of established osteosarcoma cell lines, as analyzed by flow cytometry (Figure 1), was correlated with relative CD70 gene (mRNA) expression of these cell lines, as analyzed by microarray. The regression coefficient (r^2^) between CD70 protein and CD70 gene expression was 0.87 (p = 0.002) as calculated by linear regression analysis. **(B)** CD70 relative gene (mRNA) expression in 83 biopsies of the primary tumor of 83 osteosarcoma patients depicted with the median and interquartile range. Elevated CD70 gene expression was evaluated as above the upper 25 percentile (16 patients, as indicated by the closed symbols). Asterix: biopsy L1372 of osteosarcoma patient 88 with high CD70 gene expression and available frozen tissue for immunohistochemical and immunofluorescent protein stainings as depicted in Figure 4. **(C)** Metastasis-free survival of osteosarcoma patients during ten years of follow-up comparing survival with high and low CD70 gene expression. Of the 83 patients (n = 16: CD70^high^ gene expression and n = 67: CD70^low^ gene expression) depicted in panel B, 13 patients with metastasis at time of diagnosis were excluded, resulting in the inclusion of 70 patients (n = 15: CD70^high^, and n = 55: CD70^low^) for this analysis. Mantel-Cox univariate survival analysis resulted in a log-rank χ^2^ score of 0.29 (p = 0.59).

Therefore, it was first investigated whether CD70 mRNA expression correlated with protein expression in osteosarcoma cell lines. CD70 protein expression in osteosarcoma cell lines indeed correlated with CD70 mRNA expression in these cells lines (r^2^ = 0.87, p < 0.002) (Figure 3, panel A).

**Figure 4:**
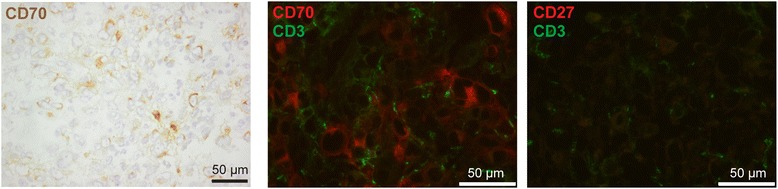
**CD70 protein expression in osteosarcoma lesions is expressed on tumor cells and not on tumor-infiltrating T cells.** Immunohistochemical stainings for CD70 and immunofluorescent double stainings for CD70 (red) and CD3 (green) as well as CD27 (red) and CD3 (green) (cell nuclei in blue) was assessed in sequential specimens of the osteosarcoma biopsy L1372 (p88) with high CD70 gene expression as indicated by the asterix in Figure 3, panel A.

Next, CD70 gene expression was evaluated in diagnostic biopsies of 83 osteosarcoma patients. A subset of patients (16/83) showed significantly higher CD70 gene expression (above the upper 25 percentile as indicated) (Figure 3, panel B). Of note, high CD70 gene expression corresponded to strong CD70 protein staining in the tumor biopsy as assessed for one patient (p88) with available frozen tissue (L1372) (Figure 4, panel A; patient (p88) indicated by * in Figure 3, panel A).

To investigate the association of CD70 expression by tumor cells with clinical outcome, we first assessed whether CD70 was expressed by tumor cells and not by tumor-infiltrating T cells in this biopsy with high CD70 gene/protein expression (L1372, p88), since activated T cells are known to express CD70 [9]. Strong immunofluorescent staining for CD70 was confined to tumor cells and did not co-localize with the T cell marker CD3 (Figure 4, panel B). In contrast to CD70, its receptor CD27 was detected neither in CD70^+^ nor CD70^−^ osteosarcoma lesions and was not expressed by infiltrating T cells (Figure 4, panel C and data not shown).

Next, it was determined whether higher CD70 gene expression was correlated with the occurrence of metastasis, which is the most important parameter for survival of osteosarcoma patients. Thirteen patients presenting with metastasis at time of diagnosis were excluded from this analysis. In the remaining 70 patients, the occurrence of overt metastasis (metastasis-free survival) during ten years of follow-up was not statistically different between patients with high or low CD70 gene expression (log-rank χ^2^ = 0.29, p = 0.59) (Figure 3, panel C). There was no association between CD70 gene expression and a specific tumor location or histological subtype (data not shown).

In conclusion, high CD70 expression in osteosarcoma tumors is not associated with better or worse metastasis-free survival.

## Discussion

We demonstrate that CD70 is expressed on the majority of osteosarcoma cell lines as well as patient-derived osteosarcoma cultures which were derived from CD70^+^ tumor tissue. CD70 expression in tumor lesions was heterogeneous and restricted to tumor cells and not attributed to infiltrating T cells. CD70 gene expression in diagnostic biopsies was significantly higher in a subset of osteosarcoma patients but this difference was not correlated with metastasis-free survival.

Among pediatric bone and soft tissue cancers, we found that CD70 is preferentially expressed on osteosarcoma cells (9/13). CD70 was detected on few Ewing sarcoma cells (5/15) and was virtually absent from neuroblastoma and rhabdomyosarcoma cells, indicating that CD70 expression is restricted to certain cancer types like renal cell carcinoma and osteosarcoma and to a lesser extent brain cancers, larynx or pharynx cancer, melanoma, pancreatic cancer, ovarian carcinoma and Ewing sarcoma [6,7,20,24-26]. Among the osteosarcoma specimens we tested, CD70 expression was heterogeneous between patients as well as within the tumor. In a larger cohort of 83 osteosarcoma patients, a subset of tumor biopsies (19%) showed significantly higher CD70 gene expression.

This raises the question whether CD70 exerts a biological function in certain cancer types or certain cancer cells. In some tumor-transformed B cells, CD70 (downstream) signaling may affect proliferation and apoptosis [19,20,27]. In spite of this, in most studies, biological effects of CD70 have been demonstrated to be mediated by interactions of CD70 with its receptor CD27, which can have a dual role in regulating anti-tumor adaptive immune responses. Acute challenge with CD70^+^ tumor cells was shown to induce anti-tumor T cell-mediated immunity [18]. Similarly, CD70-CD27 interactions promote anti-virus and anti-tumor T cell responses [28,29]. On the contrary, co-culture of CD70^+^ tumor cells and CD27^+^ immune cells was shown to inhibit alloreactive T cell proliferation and induce T cell apoptosis [20,30]. Similarly, continuous CD70-CD27 co-stimulation in CD70-transgenic mice or in mice chronically infected with mouse choriomeningitis virus exhausts the naïve T cell pool in favor of effector memory T cells that ultimately results in T cell dysfunction unless relieved from the CD70-CD27 brake [28,31]. In line with the latter observations, CD70^+^ tumor cells in renal cell carcinoma and B cell lymphoma may promote depletion of naïve CD27^+^ T cells and induce regulatory T cells in the tumor, respectively [32,33].

Hence, during a persistent tumor-immune cell interaction, CD70 expression on tumor cells may support tumor escape from immunosurveillance and thus be disadvantageous for patient outcome. However, to our knowledge, a significant association of CD70 expression on tumor cells with patient survival has not been described in these human tumors [26]. In our cohort of osteosarcoma patients, CD70 gene expression at the time of diagnosis did not correlate with metastasis-free survival, suggesting that at least in osteosarcoma, CD70 expression does not promote tumor progression. Moreover, CD27 was neither detected on tumor cells nor on infiltrating T cells in osteosarcoma lesions, suggesting that CD70 on tumor cells is not involved in CD27-dependent tumor-immune cell interactions. In spite of the ambiguous role of CD70 in cancer development and progression, its substantial expression in certain cancers would make CD70 an attractive immunotherapeutic target. This is supported by the highly restricted expression of CD70 on immune cells and its virtual absence in normal non-hematopoietic tissue, which may limit side-effects of CD70 targeted treatment [6,24]. In this perspective, chimeric antigen receptor T cells (CD27-CD3ζ) have been reported to kill CD70^+^ tumor cells and mediate tumor regression in mice [34]. Moreover, therapeutic chimeric and humanized anti-CD70 antibodies are currently developed. In preclinical studies, anti-CD70 antibodies demonstrated indirect anti-tumor efficacy mediated by NK cells and macrophages involving antibody-dependent cellular cytotoxicity and tumor cell phagocytosis, respectively, as we have similarly recently reported using the clinically-approved anti-epidermal growth factor receptor antibody cetuximab [22,35]. In addition, if conjugated to cytotoxic drugs anti-CD70 antibodies can mediate direct anti-tumor effects [6,24]. Since we had no access to chimeric or humanized anti-CD70 antibodies, we could not test direct or indirect cytotoxicity against CD70-expressing osteosarcoma cells. Altogether, in osteosarcoma patients with CD70-postive tumors, CD70 may constitute a tumor antigen for novel targeted immunotherapy.
